# The ALS-associated co-chaperone DNAJC7 mediates neuroprotection against proteotoxic stress by modulating HSF1 activity

**DOI:** 10.1101/2024.12.01.626216

**Published:** 2024-12-01

**Authors:** Andrew C. Fleming, Nalini R. Rao, Matthew Wright, Jeffrey N. Savas, Evangelos Kiskinis

**Affiliations:** 1 The Ken & Ruth Davee Department of Neurology, Feinberg School of Medicine, Northwestern University, Chicago, IL 60611, USA.; 2 Simpson Querrey Institute, Northwestern University, Chicago, Illinois 60611, USA.; 3 Department of Neuroscience, Northwestern University Feinberg School of Medicine, Chicago, IL, 60611, USA.

**Keywords:** DNAJC7, Amyotrophic lateral sclerosis (ALS), HSF1, chaperone, stress response, HSP70, motor neurons, iPSC models, HNRNPU

## Abstract

The degeneration of neurons in patients with amyotrophic lateral sclerosis (ALS) is commonly associated with accumulation of misfolded, insoluble proteins. Heat shock proteins (HSPs) are central regulators of protein homeostasis as they fold newly synthesized proteins and refold damaged proteins. Heterozygous loss-of-function mutations in the *DNAJC7* gene that encodes an HSP co-chaperone were recently identified as a cause for rare forms of ALS, yet the mechanisms underlying pathogenesis remain unclear. Using mass spectrometry, we found that the DNAJC7 interactome in human motor neurons (MNs) is enriched for RNA binding proteins (RBPs) and stress response chaperones. MNs generated from iPSCs with the ALS-associated mutation R156X in *DNAJC7* exhibit increased insolubility of its client RBP HNRNPU and associated RNA metabolism alterations. Additionally, DNAJC7 haploinsufficiency renders MNs increasingly susceptible to proteotoxic stress and cell death as a result of an ablated HSF1 stress response pathway. Critically, expression of HSF1 in mutant DNAJC7 MNs is sufficient to rescue their sensitivity to proteotoxic stress, while postmortem ALS patient cortical neurons exhibit a reduction in the expression of HSF1 pathway genes. Taken together, our work identifies DNAJC7 as a crucial mediator of HNRNPU function and stress response pathways in human MNs and highlights HSF1 as a therapeutic target in ALS.

## INTRODUCTION

Amyotrophic Lateral Sclerosis (ALS) is a devasting neurodegenerative disease which is driven by the dysfunction and degeneration of upper and lower motor neurons (MNs)^1,2^. The progressive loss of MNs leads to muscle atrophy and diminished ability to stimulate and control movement^1,2^. ALS cases are predominantly sporadic in nature, but a relatively small proportion of patients (<12%) suffers from familial forms of the disease^3^. The genetic etiology underlying these familial cases is complex, comprised of over 30 disease-causing genes encoding functionally diverse proteins involved in cellular functions ranging from RNA processing, cytoskeletal homeostasis, and axonal transport to protein quality control pathways^4–6^.

*DNAJC7* is a recently identified ALS-associated gene^7^ that is of particular interest because it encodes a co-chaperone protein with an ostensible but relatively unexplored role in protein homeostasis^8^, a process known to be perturbed in ALS and other neurodegenerative diseases^9^. It was first identified as an ALS-associated gene in a whole-exome sequencing (WES) study that highlighted a significant enrichment for protein truncating variants (PTVs) in *DNAJC7*, including nonsense and rare missense variants predicted to be pathogenic^7^. Importantly, additional disease-causative variants in *DNAJC7* have been identified in a series of subsequent independent studies on small cohorts of ALS patients^10–15^. Most ALS enriched variants in *DNAJC7* are nonsense, while many missense variants are located within functional protein domains, strongly suggesting disease mechanisms driven by DNAJC7 haploinsufficiency^16^. However, the mechanisms by which mutant DNAJC7 causes MN dysfunction and eventual degeneration remain unknown.

The DNAJC7 protein is highly expressed within the central nervous system (CNS) and belongs to a superfamily of co-chaperone proteins known as HSP40s that are responsible for maintenance of protein homeostasis processes including *de novo* protein folding and cellular stress response pathways^8,16,17^. The specific role of the HSP40s co-chaperones such as DNAJC7 is to facilitate the unambiguous selection of substrates for HSP70/HSP90 chaperones amongst the promiscuous pool of available client proteins^8,18^. As such, the known interacting partners of DNAJC7 include several protein components of the HSP family including HSP70s (e.g., HSPA4, HSPA8) and HSP90s, where it has been shown to “bridge” clients between HSP70 and 90^8,16–20^. While very little is known about the specific role or binding partners of DNAJC7 in neurons, the HSP70/90 complexes play critical roles in cellular proteostasis pathways. One such pathway is the heat shock response (HSR) pathway, which is governed by the highly conserved master transcription factor heat shock factor 1 (HSF1), and is broadly utilized by cells to cope with proteotoxic stress (e.g., stress caused by misfolded proteins) by upregulating the expression of heat shock proteins (HSPs) and chaperones^21–24^.

Here, we sought to determine how mutant DNAJC7 causes MN dysfunction. We discovered novel DNAJC7 interactors in human MNs, which were enriched for proteins participating in RNA metabolism and cellular responses to stress. Using CRISPR-Cas9 edited iPSC-derived MN models, we found that DNAJC7 haploinsufficiency disrupts mRNA metabolism through increased insolubility of HNRNPU. Most notably we found that mutant DNAJC7 MNs are sensitized to proteotoxic stress and exhibit degeneration because of a disruption in the timely activation of HSF1. Overexpression of HSF1 in mutant DNAJC7-MNs rescued their premature degeneration upon prolonged exposure to proteotoxic insult. Our findings elucidate the mechanisms by which DNAJC7 regulates MN proteostasis and highlight the potential of HSF1 stimulation as a therapeutic target in ALS to counter MN vulnerability.

## RESULTS

### DNAJC7 interacts with RNA binding proteins and regulators of stress response pathways in human MNs.

**Figure 1.**
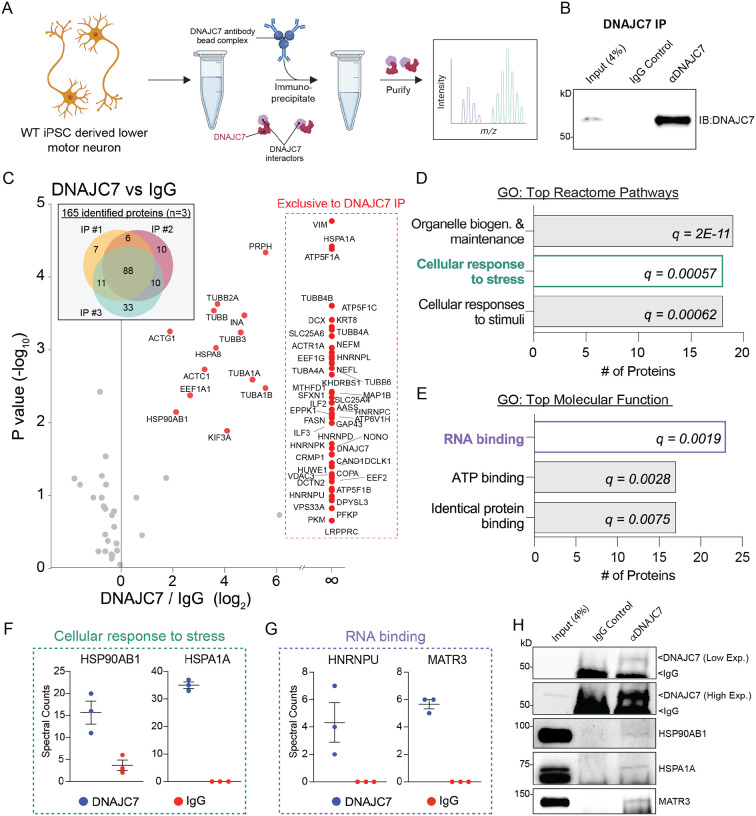
DNAJC7 interacts with RNA binding proteins and regulators of stress response pathways in human MNs. (A) Schematic of co immunoprecipitation of DNAJC7 from WT MNs. (B) WB of DNAJC7 from co-IP. Input = 4% of total protein used in co immunoprecipitation. (C) Venn diagram: all identified proteins from each DNAJC7 IP (n=3), 88 of which persistently identified proteins from each biological replicate. Scatter plot: Gray dots represent proteins not significantly enriched in DNAJC7 IP, red dots represent proteins significantly enriched in DNAJC7 IP fraction (p<0.05) or found exclusive in DNAJC7 IP (61 proteins). (D and E) Gene ontology of pathways overrepresented within DNAJC7 interactome, filtered by fold enrichment and ranked by number of proteins. FDR = q value. (F and G) Plot of spectral counts of proteins found in DNAJC7 IP vs IgG IP. Values represent the mean ± standard error of the mean (SEM). Each dot represents a biological replicate (N = 3). (H) WB of proteins co immunoprecipitated with DNAJC7. Input = 4% of total protein used in co immunoprecipitation. DNAJC7 WB: top band = DNAJC7 protein, lower band = IgG heavy chain visualized at low and high exposure. Note that IgG heavy chain does not appear in panel B because the DNAJC7 antibody was covalently linked to immunoprecipitation beads.

In order to investigate the functional role of DNAJC7 in the context of ALS we sought to identify its binding partners in human MNs, which represent the most vulnerable cell type in the disease. We used a healthy control iPSC line (line CS0002, see Methods) to generate lower MNs^25^, and performed immunoprecipitation (IP) of endogenous DNAJC7, followed by liquid chromatography tandem mass spectrometry (LC-MS/MS)-based proteomic analysis (Figure 1A–B and S1A). We conducted the IP-MS experiment using MN cultures from 3 independent differentiations and identified 88 proteins that were consistently co-purified within the DNAJC7 IP (Figure 1C). Of these, 48 proteins were found to co-purify exclusively with DNAJC7, while 13 were significantly enriched within the DNAJC7 IPs, relative to the IgG control IPs (p<0.05) (Figure 1C, Figure S1B). To add further context to the DNAJC7 interactome we performed STRING and gene ontology (GO) bioinformatic analysis, which collectively highlighted “RNA binding”, “ATP binding”, “cellular response to stress” and “cytoskeleton” as significantly enriched categories (Figure 1D–E and S1C). Importantly, these terms have been previously implicated in ALS pathogenesis in both genetic analysis and observations from disease models and post-mortem patient tissue^26–29^. We specifically identified several RNA-binding proteins (RBPs) including MATR3 and members of the family of heterogeneous ribonucleoprotein particle (HNRNP) proteins such as HNRNPK, HNRNPU, HNRNPD, HNRNPC and HNRNPA1 (Figure 1C and Table S1). Notably, genetic mutations in *MATR3* and *HNRNPA1* themselves are responsible for rare forms of ALS^30^, suggesting a converging functional interaction between independent causal genes. DNAJC7 has been previously shown to interact with another ALS-causal RBP in FUS^31^, although we did not confirm this in our unbiased proteomics analysis. The “cellular response to stress” enrichment was driven by several HSPs with chaperone activity including HSPA1A, HSPA8, and HSP90 (Figure 1C and Table S1). In fact, one of the main DNAJC7 interactors was HSPA1A, an HSP70 that is tightly linked to stress response pathways and has previously been shown to interact with DNAJC7 in multiple non neuronal cell models^8,18,19,32^. To independently validate these findings, we performed IP-Western blot (WB) analysis and probed for representative proteins from the “stress response” and “RNA binding” pathways in HSPA1A, HSP90AB1 and MATR3 respectively (Figure 1F–H). Collectively, analysis of the DNAJC7 interactome in MNs showed that it physically interacts, potentially through HSP70/90 complexes, with a small and specific set of proteins enriched for RBPs and HSPs.

### DNAJC7 haploinsufficiency disrupts HNRNPU solubility and target mRNA expression.

**Figure 2.**
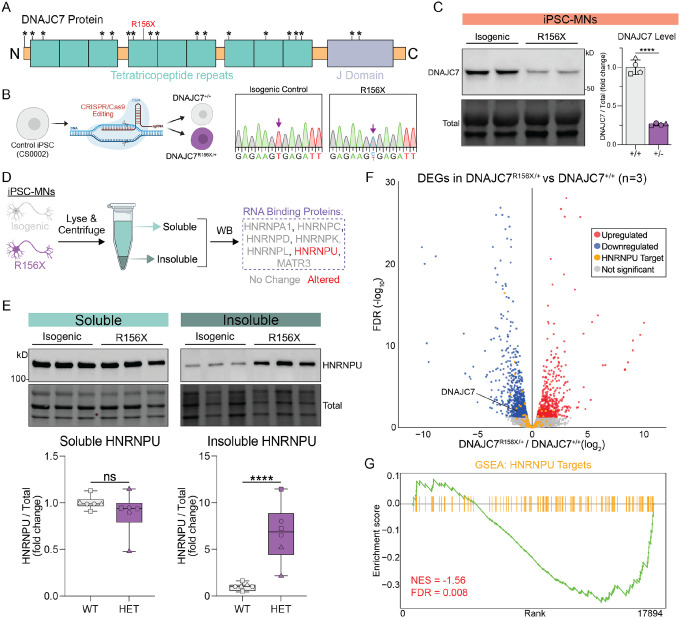
DNAJC7 haploinsufficiency disrupts HNRNPU solubility and target mRNA expression (A) Schematic of known ALS causative mutations on DNAJC7 protein marked with *. (B) Schematic of CRISPR-Cas9 knock-in of R156X mutation into healthy control iPSC line. (C) Left: WB images of DNAJC7 proteins levels from MN lysate derived from isogenic pairs (+/+ = isogenic control, +/− = R156x mutant). Bottom: Values represent the mean ± standard error of the mean (SEM). Four experiments are represented by distinct shaped symbols. N = 4, Unpaired t test (two-tailed): **** P<0.0001. (D) Schematic of biochemical fractionation of soluble/insoluble material from MN lysates followed by WB analysis. Red labels represent proteins whose solubility change in R156X mutant MNs. (E) Top: WB images of soluble and insoluble HNRNPU proteins levels from MN lysate derived from isogenic pairs. Bottom: Values represent the mean ± min/max. Experiments are represented by distinct shaped symbols. N = 6, Unpaired t test (two-tailed): **** P<0.0001. (F) Volcano plot of RNA Sequencing data from R156X vs control. Red or blue colored dots represent transcript significantly upregulated or downregulated, respectively (n = 3, FDR < 0.05). Yellow dots represent transcriptional targets of HNRNPU. (G) Gene set enrichment of HNRNPU eCLIP targets. Kolmogorov–Smirnov test: N = 3, NES = −1.56, FDR = 0.008.

We next sought to develop a cellular model to investigate the functional ramifications of ALS-associated DNAJC7 mutations (Figure 2A). We used CRISPR-Cas9 to knock-in a premature truncation point mutation (p466 C>T [R156X]) into the endogenous gene locus in a healthy control iPSC stem cell line (line CS0002, see Methods) (Figure 2B). We targeted this mutation out of more than 15 that have been reported so far (Figure 2A) because is the most common ALS-causing variant^7,16^, and it is predicted to be deleterious. The editing generated an isogenic pair of iPSC clones with two distinct genotypes: a heterozygous clone containing one copy of the mutation (DNAJC7^R156X/+^), and a targeted but unedited isogenic wild type (WT) control (Figure 2B). WB analysis showed that the DNAJC7 protein was reduced by ~75% in heterozygous mutant iPSC-derived MNs (p<0.0001), consistent with previous work demonstrating a similar level of DNAJC7 reduction in ALS patients harboring 1 copy of R156X^7^ (Figure 2C). Importantly, DNAJC7 haploinsufficiency did not impede the efficiency of motor neurogenesis, as the mutant DNAJC7 and isogenic control iPSC lines exhibited equal efficiency of differentiation as measured by immunofluorescence (IF) for the MN markers ISL1/2 and MAP2 (Figure S2A–C).

Given the potential role of DNAJC7 in *de novo* protein folding, we next sought to utilize this isogenic iPSC platform to determine if DNAJC7 client RBPs have increased insolubility, indicating potential misfolding and subsequent dysfunction (Figure 2D). Intriguingly, we found that while most RBPs were not affected, the level of HNRNPU within the insoluble biochemical fraction, was significantly higher in mutant DNAJC7 MNs relative to isogenic controls (p<0.0001) (Figure 2E). Given HNRNPU’s relatively promiscuous role as an RNA binding protein, we next performed RNA-Sequencing (RNA Seq) in differentiated MNs to capture any potential HNRNPU-dependent changes in gene expression downstream of this altered solubility. We found ~1600 differentially expressed genes in mutant DNAJC7 MNs (FDR < 0.05), and upon performing gene set enrichment analysis (GSEA) on known HNRNPU target mRNAs within the dataset, we found that they were significantly under-represented in DNAJC7^R156X/+^ cultures (normalized enrichment score [NES] = −1.56, FDR = 0.008) (Figure 2F–G, Figure S2D). As a negative control, we also performed GSEA on mRNAs associated with other RBPs (e.g., MATR3, HNRNPL) whose solubility was unaffected in mutant DNAJC7 MNs and found no significant enrichment (Figure S2E–G). Together, these data demonstrate that ALS-associated DNAJC7 haploinsufficiency impacts the solubility of HNRNPU and the expression level of some of its client mRNAs.

### DNAJC7 haploinsufficiency sensitizes MNs to proteotoxic stress.

**Figure 3.**
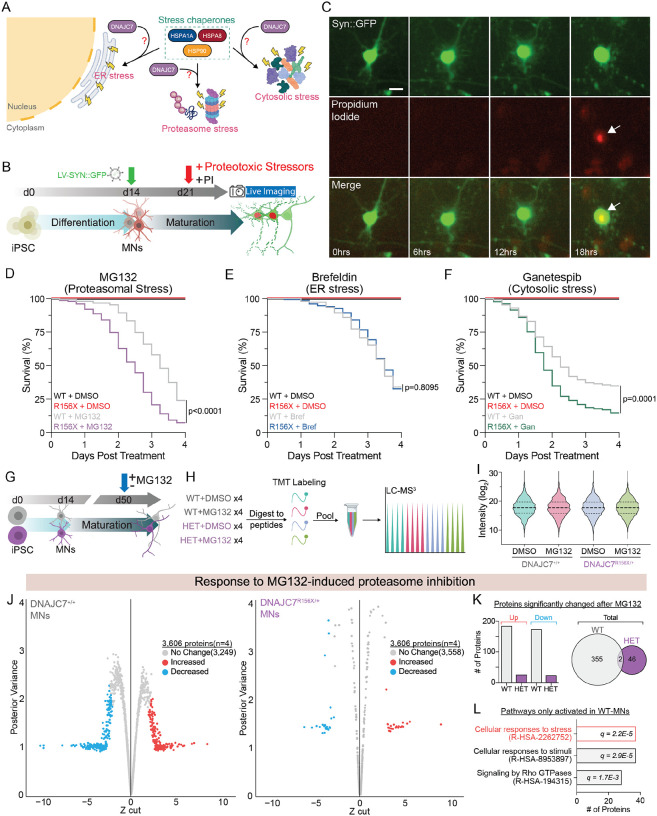
DNAJC7 haploinsufficiency sensitizes MNs to proteotoxic stress. (A) Schematic of stress pathways DNAJC7 may influence. (B) Schematic of live imaging experiments following proteotoxic stress induction. (C) Representative images of a dying MN co-labeled with SYN1-GFP (green) and propidium iodide (red) and merged. Scale bar, 20 μm. (D) Kaplan-Meier survival curve of MNs survival following MG132 or DMSO control. 150 cells tracked per condition, Mantel-Cox log-rank test: p<0.0001. (E) Kaplan-Meier survival curve of MNs survival following Brefeldin or DMSO control. 150 cells tracked per condition, Mantel-Cox log-rank test: p=0.8085. (F) Kaplan-Meier survival curve of MNs survival following Ganetespib or DMSO control. 129 cells tracked per condition, Mantel-Cox log-rank test: p=0.0001. (G and H) Schematic of MG132 treatment in MNs followed by tandem mass tag quantitative proteomics. (I) Violin plot of the average TMT intensities of all proteins for each condition. Two-way ANOVA: no significance. (J) Shrinkage plots of protein level changes in MNs following MG132 treatment for R156X-MNs (right) and isogenic controls (left). Red and blue dots represent significantly increased or decreased proteins, respectively. N = 4 independent differentiations. (K) Left: bar graph displaying number of proteins changed in response to MG132 for each genotype. Right: Venn diagram showing lack of overlap of altered proteins. (L) GO enrichment of top reactome pathways upregulated in isogenic MNs following MG132 ranked based on # of genes in pathway. FDR = q value indicated in each bar.

We next focused on dissecting the significance of DNAJC7 haploinsufficiency in cellular stress response pathways. Several chaperones including HSPA1A, HSPA8, and HSP90 that we found to bind DNAJC7 in MNs are involved in the handling of proteotoxic stress (Figure 3A). Thus, we hypothesized that loss of DNAJC7 might disrupt the ability of MNs to respond to proteotoxic stress and make them more sensitive to various relevant insults (Figure 3A). To test this hypothesis, we treated DNAJC7^R156X/+^ and isogenic control MNs with a series of small molecules that trigger endoplasmic reticulum (ER) stress (Brefeldin, 100nM), proteasomal stress (MG132, 5μM) and cytoplasmic stress (Ganetespib, 150nM) and monitored neuronal survival by live cell imaging (Figure 3B). To track the survival of individual cells we used a neuron-specific fluorescent reporter (*SYN1*::GFP) in combination with the cell death indicating dye propidium iodide (PI) (Figure 3B) and acquired single-cell resolution images of labeled MNs every 6 hours for 4 days after treatment (Figure 3C). We found that mutant DNAJC7 MNs degenerated significantly faster compared to their isogenic controls when exposed to MG132 (p<0.0001) and Ganetespib (p=0.0001), but not Brefeldin (p=0.8095), suggesting this increased sensitivity was relatively specific (Figure 3D–F). Importantly these effects were robust across n=3 independent differentiations (Figure S3A–C), while we did not observe any spontaneous degeneration after vehicle DMSO treatment in either genotype. Although the specific mechanism of action of MG132 and Ganetespib is different (MG132 blocks the proteosome and Ganetespib inhibits HSP90 activity), a notable converging consequence of both molecules is an accumulation of excess proteins^33–35^, suggesting that DNAJC7 haploinsufficiency renders MNs vulnerable to the toxic accumulation of proteins.

We next used tandem mass tag mass spectrometry (TMT-MS) to compare the proteomic response of heterozygous DNAJC7^R156X/+^ and isogenic control MNs following 8 hours of MG132 treatment, as this stressor elicited a very robust survival phenotype (Figure 3G). TMT-MS employs multiplexed isobaric labeling that confers the advantage of enabling the pooling of all biological replicates, thereby eliminating batch variability and facilitating a highly quantitative method by which to directly compare the relative abundance of proteins from the same pool of identified peptides (Figure 3H). We quantified 3,606 proteins and critically found that the labeling efficiency was equal across all biological replicates and experimental conditions (Figure 3I). While most proteins were not alerted 8 hours after blocking the proteosome, 357 proteins, corresponding to approximately 9.9% of the quantified proteome, exhibited a significant change in their abundance in control MNs (Figure 3J–K and Figure S3D). In stark contrast, only 48 proteins or only 1.3% of the quantified proteome, exhibited measurable changes in mutant DNAJC7 MNs subjected to the same treatment (Figure 3J–K and Figure S3D). Notably, the proteins that become elevated in control MNs are enriched for pathways associated with “cellular responses to stress” suggesting a lack of such a canonical and potentially protective response in mutant MNs (Figure 3L). This robust lack of proteome remodeling in DNAJC7^R156X/+^ MNs is in accordance with their increased rates of degeneration in response to proteotoxic stress.

### DNAJC7 haploinsufficiency confers a basal reduction in HSF1 signaling in MNs.

**Figure 4.**
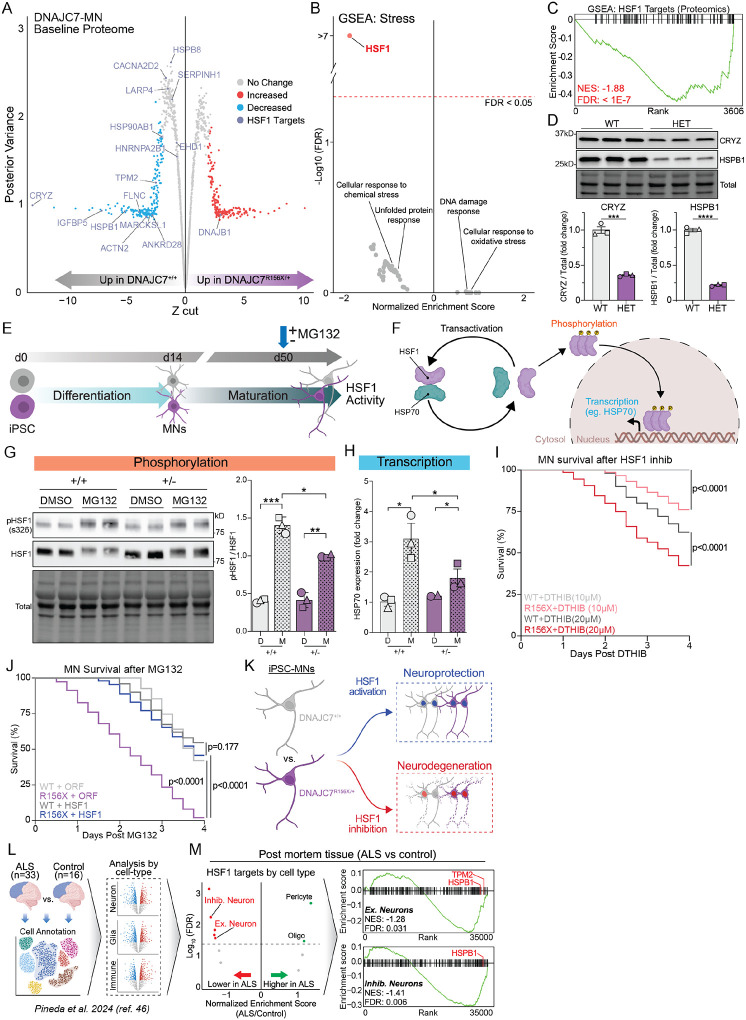
DNAJC7 regulates stress-induced activation of HSF1 and activation of HSF1 rescues the sensitivity of mutant DNAJC7-MN to stress (A) Shrinkage plot of basal relative protein abundance between DNAJC7-MNs and isogenic controls. Purple dots represent HSF1 transcriptional targets. (B) Scatter plot of gene set enrichment analysis of “stress” terms. Colored or gray dots represent significantly (FDR < 0.05) or not significantly (FDR > 0.05) enriched terms, respectively. (C) GSEA plot of HSF1 targets within the basal proteomic dataset. Kolmogorov–Smirnov test: NES = −1.88, FDR < 1E-7. (D) Top: WB image of CRYZ and HSPB1 proteins levels from MN lysate derived from isogenic pairs at baseline. Bottom: Values represent the mean ± standard error of the mean (SEM). Experiments are represented by distinct shaped symbols. N = 3, Unpaired t test (two-tailed): CRYZ ***P=0.0002, HSPB1 ****P<0.0001. (E) Schematic of HSF1 activity assessment in MNs following MG132. (F) Schematic of HSF1 activation pathway. (G) Left: WB image of phospho-HSF1 (Ser326) and total HSF1 protein levels in MNs with and without MG132. Right: Values represent ratio of pHSF1/total HSF1 ± standard error of the mean (SEM). Experiments are represented by distinct shaped symbols. N = 3, Two-way ANOVA: *p<0.05, **p<0.01, ***p<0.001. (H) RT-qPCR quantification of HSP70 transcript levels, fold change relative to WT + DMSO control. Experiments are represented by distinct shaped symbols. N = 2–3, Two-way ANOVA: *p<0.05. (I) Kaplan-Meier survival curve of MNs survival following 10 μM or 20 μM DTHIB. 144 cells tracked per condition, Mantel-Cox log-rank test: WT vs R156X (10 μM) p<0.0001, WT vs R156X (20 μM) p<0.0001. (J) Kaplan-Meier survival curve of MNs survival following MG132 with ORF-LV or HSF1-LV. 150 cells tracked per condition, Mantel-Cox log-rank test: WT+ORF vs R156X+ORF p<0.0001, R156X+HSF1 vs R156X+ORF p<0.0001, R156X+HSF1 vs WT+HSF1 p=0.177. (K) Schematic of DNAJC7-MN increased stress resistance following HSF1 activation and selective degeneration after HSF1 inhibition. (L) Schematic of single cell RNA-Seq dataset from Pineda et al.,^46^ used for analysis. (M) Left: scatter plot of gene set enrichment analysis of HSF1 Targets (MSigDB#: M19734) by cell type. Each dot represents a different cell type. Colored or gray dots represent significant (FDR < 0.05) or not significant (FDR > 0.05) in ALS vs Control samples. Inhib = Inhibitory, Ex = Excitatory, Oligo = Oligodendrocyte. Right: GSEA plots of HSF1 Targets (MSigDB#: M19734) in ALS vs Control in excitatory neurons or inhibitory. Labeled ticks indicate specific HSF1 targets also significantly reduced in DNAJC7-MNs. Kolmogorov–Smirnov test: Ex. (NES = −1.28, FDR = 0.031), Inhib. (NES = −1.41, FDR = 0.006).

We next sought to better understand the molecular mechanisms driving the increased sensitivity of mutant DNAJC7 MNs to proteotoxic stress. We first carefully examined baseline gene and protein expression differences between DNAJC7^R156X/+^ and isogenic control MNs with a focus on stress-associated terms. We identified 398 significantly altered proteins between the two genotypes and observed that several of the proteins that were significantly downregulated in DNAJC7^R156X/+^ MNs represented known targets of the heat shock transcription factor 1 (HSF1) (Figure 4A). Accordingly, a targeted gene set enrichment analysis (GSEA) of 47 unique terms directly related to “stress”, revealed a highly significant downregulation of HSF1 target proteins (FDR<0.0001) (MSigDB#: M19734) (Figure 4B–C). Critically, this effect was highly specific, as no terms associated with other types of stress such as DNA damage response (MSigDB#: M13636), unfolded protein response (MSigDB#: M5922), cellular response to oxidative stress (MSigDB#: M45123), or cellular response to chemical stress (MSigDB#: M29264) were significantly changed in mutant DNAJC7 MNs (Figure S4A). Two of the most downregulated HSF1 target proteins in the MS dataset were CRYZ (p=0.0002) and HSPB1 (p<0.0001) (Figure 4A), and we validated the reduction in their levels by WB in independently differentiated MN samples from DNAJC7^R156X/+^ and isogenic control iPSC lines (Figure 4D). GSEA on RNA-Seq datasets revealed a moderate but significant downregulation in the expression of HSF1 target genes in mutant DNAJC7 MNs (FDR = 0.02) (Figure S4B), further validating the effects on this pathway at both the transcriptional and proteomic level.

HSF1 is a highly conserved transcription factor that acts as a master regulator of cellular stress response, by activating a transcriptional cascade of chaperones, foldases and metabolic genes to counter proteotoxic stress^21–24^. Thus, a reduction in HSF1 signaling in heterozygous DNAJC7^R156X/+^ MNs may be central to their increased vulnerability to proteotoxicity. Notably, HSPA1A and HSPA8, two proteins which we found to interact with DNAJC7 in MNs, are known to play a direct role in regulating HSF1 activity^24,36,37^. The activation of this pathway has been previously associated with ALS pathophysiology, which is characterized by the accumulation of misfolded and aggregated proteins such as SOD1, FUS and TDP-43^38–43^. To add further context to the significance of HSF1 signaling for neuronal homeostasis we used publicly available single-cell RNA Seq datasets^44,45^ to examine the expression of HSF1 and its target mRNAs within distinct cell types in the human CNS (Figure S4C–D). We found that all neurons in the cortex and spinal cord including spinal MNs, expressed higher levels of HSF1 targets relative to glia, vascular and immune cells within the CNS, suggesting a potential reliance of this pathway for neuronal homeostasis.

### DNAJC7 haploinsufficiency delays stress-induced activation of HSF1.

Given the converging evidence pointing to reduced baseline HSF1 signaling, we next interrogated the molecular activation of this pathway in mutant DNAJC7 and isogenic control MNs in response to stress (Figure 4E). Activation of the HSF1 pathway consists of several well-defined steps including a dynamic coupling/decoupling of HSF1 with HSP70, HSF1 hyperphosphorylation, nuclear translocation and ultimately transcriptional upregulation of stress-response target genes (Figure 4F). We first measured the level of phosphorylated HSF1 (Ser326), a marker of active HSF1, and found that while both sets of MNs showed increased phosphorylation after MG132 treatment, the ratio of pHSF1 over total HSF1 was significantly reduced in DNAJC7^R156X/+^ MNs (n=3; p=0.033) (Figure 4G). To directly measure stress-dependent HSF1 activity we used RT-qPCR to evaluate the transcriptional level of the canonical HSF1 target *HSP70* and found reduced upregulation in mutant DNAJC7 MNs (n=3; p=0.0107) (Figure 4H). These data suggest that DNAJC7 haploinsufficiency in MNs disrupts the timely activation of HSF1 upon induction of proteotoxic stress.

### Activation of HSF1 rescues the sensitivity of mutant DNAJC7-MN to stress.

To directly determine the contribution of HSF1 signaling to the vulnerability of mutant DNAJC7 MNs we next evaluated the effect of inhibiting or activating this pathway on neuronal survival. We first treated mutant and wildtype MNs with the highly specific HSF1 inhibitor DTHIB, which acts by binding to the DNA-binding domain of HSF1 and performed longitudinal live cell imaging analysis as described before. At low doses of treatment (10μM), DNAJC7^R156X/+^ MNs demonstrated a ~25% reduction in survival, whereas isogenic control MNs were virtually unaffected with only <1% reduction in survival (n=3; p<0.0001) (Figure 4I). Similarly, treatment with a higher dose of DTHIB (20μM) caused a substantially larger reduction in the survival of DNAJC7^R156X/+^ MNs (61%) relative to its effect in control MNs (40%) (n=3; p<0.0001) (Figure 4I), demonstrating a dose-dependent sensitivity of MNs to this molecule, which is dramatically heightened by DNAJC7 haploinsufficiency.

We next used a lentiviral vector to infect MG132-treated MNs with HSF1 (HSF1-LV) or an empty vector as a control (ORF-LV). We ensured that the overexpression level of HSF1 was equal across both genotypes (Figure S4F), and that the empty vector did not have any effect on neuronal survival (Figure S4E). As we observed previously (Figure 3D–F), mutant DNAJC7 MNs were substantially more vulnerable to proteasomal inhibition than controls (WT-ORF *vs*. R156X-ORF; n=3, p<0.0001) (Figure 4J). Remarkably an only 1.5-fold level of HSF1 overexpression selectively improved the survival of mutant DNAJC7 MNs by 40% (R156X-ORF *vs*. R156X-HSF1; n=3, p<0.0001) (Figure 4J). The effect of HSF1 was minor in isogenic control MNs, resulting in no quantifiable differences in survival between the two groups of HSF1-treated MN genotypes (WT-HSF1 *vs*. R156X-HSF1; n=3, p=0.177) (Figure 4J). Taken together, our results demonstrate that mutant DNAJC7-MNs *in vitro* are particularly sensitive to HSF1 modulation, with overexpression being neuroprotective and inhibition causing neurodegeneration (Figure 4K).

Lastly, given that our findings revealed a bidirectional sensitivity of mutant DNAJC7 MNs to HSF1 modulation, we investigated whether the HSF1 pathway is relevant in the context of sporadic ALS disease. We used a single-cell RNA Seq dataset^46^ derived from human post-mortem cortical tissue comparing ALS patients (n=33, sporadic and mutant C9orf72) to age-matched healthy controls (n=16) (Figure 4L). We employed a “pseudo-bulk” analysis pipeline to measure cell-type-specific effects in ALS patient and control samples and found a significant reduction in the expression of HSF1 target genes in ALS inhibitory neurons (FDR = 0.006) and excitatory neurons (FDR = 0.031) relative to controls (Figure 4M, left). Moreover, targeted GSEA showed a significant downregulation of HSF1 pathway genes in cortical ALS neurons including HSPB1 (Figure 4M, right), analogous to the transcriptomic (Figure S4B) and proteomic (Figure 4C) profiles of iPSC-derived DNAJC7-MNs. This analysis suggests that HSF1-associated dysfunction may be a broadly pervasive feature of ALS.

## DISCUSSION

HSPs play a crucial role in the proteostasis deficits that pervade ALS pathophysiology. However, how loss of DNAJC7, which represents the only genetically linked HSP to ALS, causes MN dysfunction had remained unknown. To address this gap in knowledge, we identified DNAJC7 interacting partners and developed human iPSC-based models to interrogate functional defects in DNAJC7-deficient spinal MNs. We found that DNAJC7 haploinsufficiency reduced the solubility of the RBP HNRNPU and caused alterations in associated target mRNAs. We also found that DNAJC7 haploinsufficiency reduced the basal activity of HSF1 and impeded the timely activation of the HSF1 pathway in response to proteotoxic stress. These defects, which caused substantial vulnerability of MNs to proteotoxic stress, could be rescued by mild HSF1 overexpression. Our findings establish an indispensable role for HSF1 signaling in MN homeostasis, demonstrate that DNAJC7-associated sensitivity to stress is driven by a disruption in HSF1 activation, and highlight the therapeutic potential of targeting HSF1 to combat MN degeneration.

Intriguingly, we found a strong enrichment of RBPs that associate with DNAJC7 in human MNs including two proteins that can cause rare forms of ALS when mutated^30^, MATR3 and HNRNPA1, suggesting a converging interaction. We focused on HNRNPU because we found clear evidence for increased insolubility for this protein in mutant DNAJC7 MNs, at least under basal conditions. HNRNPU is an RBP that targets multiple RNAs to regulate their splicing and expression and plays a key role in cortical development^47^, while mutations in *HNRNPU* have been associated with rare forms of pediatric neurodevelopmental epilepsy^48^. The role if any at all, of HNRNPU in ALS pathophysiology has not been extensively explored, although it has been shown to directly bind to TDP-43 in nucleus and modulate TDP-43-dependent splicing and neurotoxicity^49^. It has also been flagged as an RBP with strong relevance to ALS by AI-based analysis of gene expression datasets and published literature^50^. While we found a disruption in the HNRNPU target gene expression signature in DNAJC7^R156X/+^ MNs, our work did not explore the functional relevance of these alterations or the potential implications of this phenotype in the context of sporadic ALS disease.

Beyond RBPs the DNAJC7 interactome analysis we conducted in MNs identified several components of cytoskeletal homeostasis including the microtubule proteins TUBA1B and TUBA4A. Although we did not observe any overt defects in relevant pathways such as neuronal morphology in our *in vitro* models of DNAJC7, cytoskeletal defects have been directly implicated in other forms of genetic ALS as well as sporadic disease^29,51,52^. Interestingly, recent studies have highlighted a functional interaction between DNAJC7, and the microtubule associated protein tau that is encoded by *MAPT*^53,54^. DnaJC7 was shown to coprecipitate with soluble human tau from brain preparations of a tauopathy mouse model and displayed higher binding affinity to natively folded WT relative to mutant tau, suppressing tau aggregation^53^. In a separate study, DNAJC7 was purified with insoluble mutant tau in HEK-293 cells, and subsequent knockdown of DNAJC7 both decreased the clearance of aggregated tau and exacerbated the seeding of tau *in vitro*^54^. Interestingly, overexpression of WT DNAJC7 abrogated mutant tau seeding, while introduction of ALS-associated mutations on the J-Domain of *DNAJC7*, which mediates interaction with HSP70, nullified this rescue. Tau aggregates are seen in as much as 50% of frontotemporal dementia (FTD) patients^55^, a neurological disorder characterized by speech and executive dysfunction that shares genetic and pathological overlap with ALS, while 50% of ALS patients are also characterized by FTD symptoms. Although we did not specifically identify tau in our native human MN models, these studies^53,54^ flag another potential neurodegenerative disease-relevant function of DNAJC7. Additionally, it is currently unknown whether ALS patients with *DNAJC7* mutations exhibit any tau pathology and whether mutations in *DNAJC7* can cause FTD-TAU. The continuous characterization of DNAJC7 patients and expanding ALS/FTD genetic studies will provide important clarity.

Our findings on DNAJC7 are in strong accordance with multiple studies that have demonstrated the neuronal protection qualities of other DNAJ co-chaperones in ALS, FTD, and Parkinson’s disease models, highlighting the converging importance of this class of proteins in neurodegenerative disease through their critical role in neuronal proteostasis^53,56–60^. In accordance with the canonical role of DNAJ co-chaperones we found that DNAJC7 was associated with several other HSPs, including HSP70s and HSP90s, which are involved in the modulation of cellular stress responses^36,37,61–63^. Consequently, we found DNAJC7-deficient MNs were highly sensitive to proteotoxic stress induced by small molecule treatments including MG132, which causes a buildup of proteins by blocking the proteasome, and Ganetespib that leads to a similar toxic accumulation of proteins by decoupling HSP90 from its clients^34,35^. These molecules undoubtedly trigger several stress pathways, but we focused on HSF1 based on unbiased gene and protein expression data supporting a DNAJC7-associated reduction in the levels of HSF1 associated chaperones both at basal conditions, as well as after stress induction. The HSR is a highly conserved proteostasis response and forms a cyclical regulatory network with several HSPs including DNAJC7 and its interacting partners such as HSP70 and HSP90^22,62^. HSF1 is a master transcription factor that elicits the HSR cascade by binding to heat shock elements (HSEs) on the promoter regions of heat shock responsive genes and activates their expression^21–23^. HSP70 and HSP90 are two such HSR genes, and upon sufficient protein expression act to suppress the activity of HSF1, forming a closed loop of autoregulation^36,37,61–63^. We provide experimental evidence supporting a key regulatory role for DNAJC7 in the timely activation of HSF1, likely through its co-chaperone activity on HSP70 and HSP90.

The canonical transactivation of the HSF1 pathway follows a cyclical HSP70-titration model and is thought to be driven by the dynamic coupling/uncoupling of the inhibitory HSP70/HSF1 complex^24^. This on/off chaperone switch is finely tuned^37^, and any disruptions in its cycling render the acute HSF1 stress response largely inadequate^36^. We found that iPSC-MNs show high expression levels of established HSF1 target genes such as HSPB1 and CRYZ, which were downregulated in mutant DNAJC7 MNs. These findings were also supported by our analysis of gene expression in CNS tissues that showed healthy control cortical neurons and spinal MNs distinctly favoring high HSF1 signaling. These data suggest that the pathway might already be active in MNs. We have similarly previously shown that iPSC-MNs as well as human spinal cord tissue neurons exhibit high basal levels of ER-stress as measured by XBP1-splicing^64^. It may be that post mitotic cells such as MNs constitutively utilize these stress pathways in order to cope with accumulated proteostasis insults over their especially long lifespan^65,66^. In this context, a DNAJC7-dependent disruption in HSF1 signaling would sensitize MNs to age-associated stress. At the same time, mouse MNs have previously been shown to maintain a high threshold of induction of the HSF1-mediated stress response relative to other cell types including glial cells^67^, with the suggestion that this contributes to their vulnerability to stress signals such as insoluble proteins. How exactly DNAJC7 depletion disrupts HSF1 activation remain to be determined but given its strong interaction with HSP70 and HSP90 it likely disrupts the HSF1 transactivation complex.

Our work highlights the HSF1 pathway as a therapeutic target in ALS patients with DNAJC7 mutations, and perhaps beyond^68^, as RNA-Seq analysis revealed a reduction in the expression of HSF1 pathway genes in sporadic and C9orf72 patient neurons. There have been several notable associations between ALS pathophysiology and dysfunction in elements directly downstream of HSF1 activation in response to stress, including accumulation of misfolded and aggregated proteins such as mutant FUS, SOD1 and OPTN^38–41^, perturbed HSP-mediated autophagic clearance of TDP-43^42,43^, and reduced expression of HSP40 and HSP70 in sporadic ALS spinal cord tissue^26^. Notably, HSF1 has been shown to prolong the survival of a mutant SOD1-ALS mouse model^69^, while *Chen et al*., found evidence of a compromised HSF1 response pathway in transgenic TDP-43 mouse models and ALS patients, and showed that a constitutively active form of HSF1 reduced the level of insoluble TDP-43 in mammalian cells^26^. Similarly, studies have shown that both FUS and C9ORF72 mutations trigger the HSF1 stress response pathway as an early means of neuronal protection, further illustrating the pervasive relevance of the timely activation of HSF1 in ALS^70,71^. The therapeutic potential of HSF1 is further bolstered by the finding that Riluzole, an FDA-approved drug that has a moderate but reproducible effect on extending ALS patient survival^72^, is thought to at least in part, elicit its benefit by increasing latent HSF1 levels^73^. At the same time, a phase 3 clinical trial in ALS patients using Arimoclomol^74^, which is a related HSP70 inducer that had shown promise in preclinical mouse models, was terminated as it did not lead to measurable efficacy outcomes^75,76^. HSF1 stimulation may be a challenging target with a narrow therapeutic index as most available molecules, agonize the pathway by causing mild stress^68^. A better understanding of the mechanisms by which HSF1 stimulation is protective in human MNs might allow for the design of more specific and safer molecules. Lastly, our findings underscore the importance of conducting a comprehensive characterization of this pathway and its specific gene targets in human MNs, which likely require a high basal level of HSF1 signaling for their homeostasis.

### Limitations of the study

While iPSC-models offer some unique advantages related to the ability of study human neurons, they are also limited on account of the fact that they represent *in vitro* models, without the context of an intact nervous system of a model organism. Additionally, our work is focused exclusively on the impact of DNAJC7 loss-of-function effects on spinal-like MNs, which are the cells that are primarily affected in ALS disease pathogenesis, but any potential non-cell autonomous effects remain unexplored. Lastly, while we show that HSF1 overexpression substantially ameliorates proteotoxic neurodegeneration of mutant DNAJC7 MNs, we did not define the mechanistic effects of HSF1 activation. It remains to be determined whether other genes that act downstream of HSF1 such the Bcl2-associated athanogene 3 (BAG3)^77^, a pro-survival co-chaperone to HSP70, would act in a similar way to protect mutant DNAJC7 MNs.

## Supplementary Material

1
