# Supplementary material for: The ALS-associated co-chaperone DNAJC7 mediates neuroprotection against proteotoxic stress by modulating HSF1 activity
Source: bioRxiv. 2024 Dec 1:2024.12.01.626216. Preprint. [Version 1] doi: 10.1101/2024.12.01.626216 (PMC11623670; doi:10.1101/2024.12.01.626216)
Supplement: 1 [file NIHPP2024.12.01.626216V1-supplement-1.pdf]

bioRxiv preprint doi: https://doi.org/10.1101/2024.12.01.626216; this version posted December 1, 2024. The copyright holder for this preprint (which was not certified by peer review) is the author/funder, who has granted bioRxiv a license to display the preprint in perpetuity. It is made available under aCC-BY 4.0 International license.

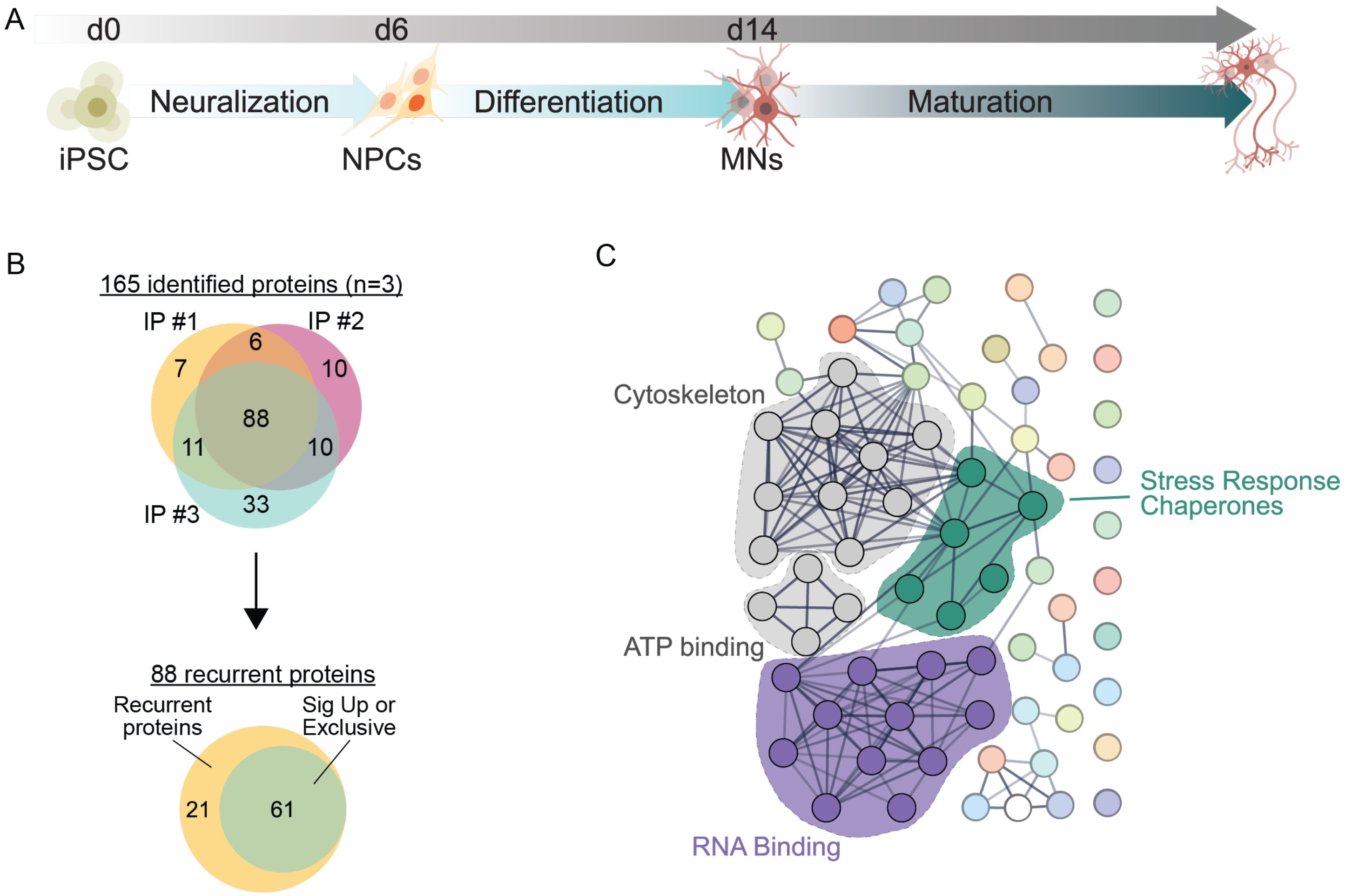

Figure S1.

(A) Schematic of 14-day small-molecular iPSC differentiation to lower motor neurons.

(B) Venn diagram of 88 proteins redundantly identified across n = 3 experiments. Of those 88, 61 proteins were significantly enriched in DNAJC7 IP or exclusively identified within the DNAJC7 IP.

(C) STRING analysis of DNAJC7 interactome. Colors correspond to relevant identified GO enrichment pathways.

bioRxiv preprint doi: https://doi.org/10.1101/2024.12.01.626216; this version posted December 1, 2024. The copyright holder for this preprint (which was not certified by peer review) is the author/funder, who has granted bioRxiv a license to display the preprint in perpetuity. It is made available under aCC-BY 4.0 International license.

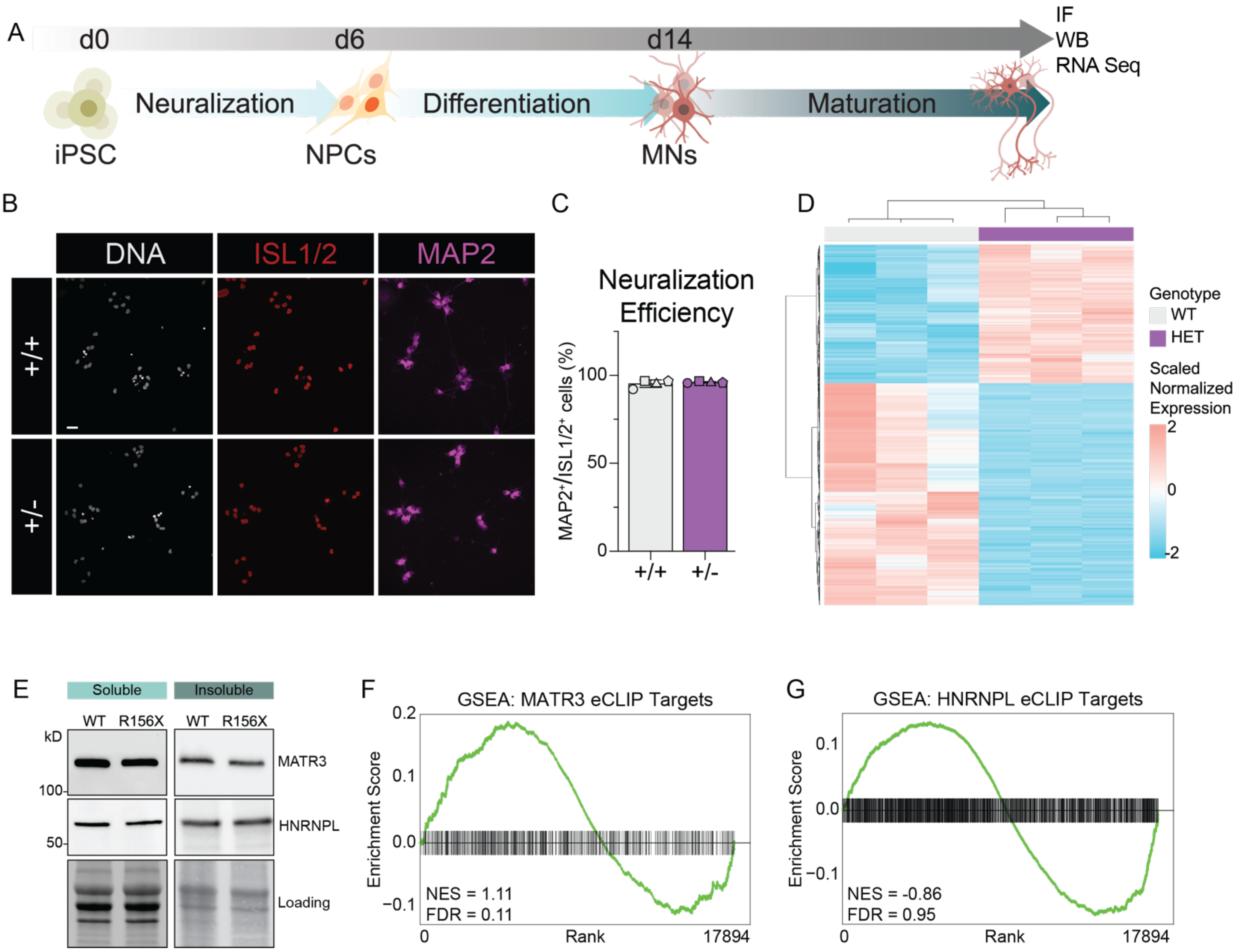

Figure S2. DNAJC7 haploinsufficiency disrupts HNRNPU solubility and target mRNA expression

(A) Schematic of iPSC differentiation into lower motor neurons followed by immunofluorescence (IF), WB or RNA Sequencing following maturation of 50 days in culture.

(B) Confocal images of MNs derived from DNAJC7 isogenic pair immunolabeled with anti ISL1/2, anti MAP2, and Hoechst 33342. Scale bar, 25 µm.

(C) Quantification of B, values represent the mean ± standard error of the mean (SEM). Experiments are represented by distinct shaped symbols. N = 4.

(D) Heat map of differentially expressed genes (FDR < 0.05) in RNA Seq of R156X vs isogenic control, N = 3.

(E) WB images of soluble and insoluble MATR3 and HNRNPL proteins levels from MN lysate derived from isogenic pairs.

(F and G) Non-significant gene set enrichment of MATR3 and HNRNPL eCLIP targets (Kolmogorov–Smirnov test).

bioRxiv preprint doi: https://doi.org/10.1101/2024.12.01.626216; this version posted December 1, 2024. The copyright holder for this preprint (which was not certified by peer review) is the author/funder, who has granted bioRxiv a license to display the preprint in perpetuity. It is made available under aCC-BY 4.0 International license.

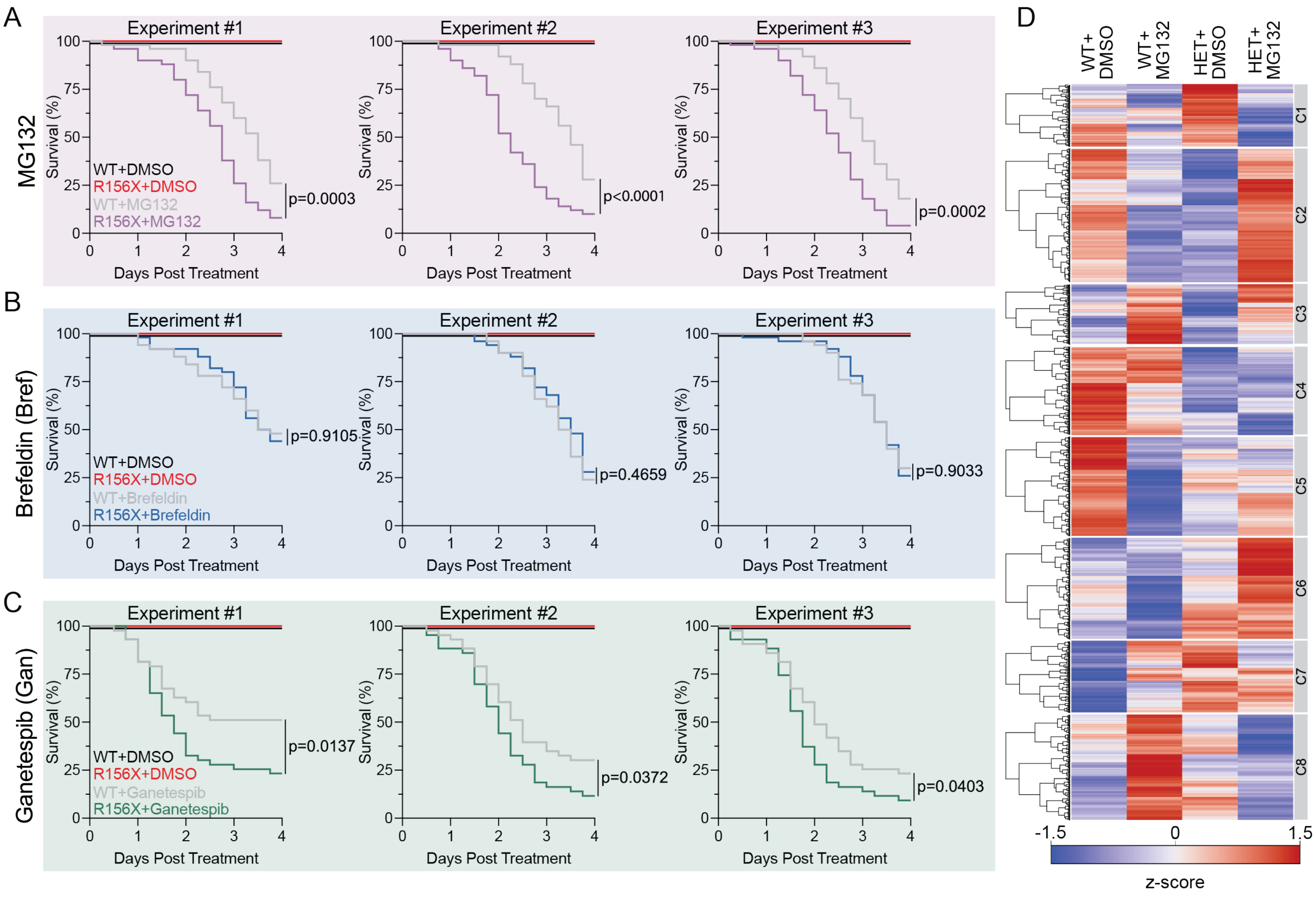

Figure S3. DNAJC7 haploinsufficiency sensitizes MNs to proteotoxic stress.

(A) Kaplan-Meier survival curve of MNs survival following MG132 or DMSO control. Three independent differentiations separated. 50 cells per condition per experiment, Mantel-cox log-rank test: $p=0.0003$, $p<0.0001$, $p=0.0002$.

(B) Kaplan-Meier survival curve of MNs survival following Brefeldin or DMSO control. Three independent differentiations separated. 43 cells per condition per experiment, Mantel-cox log-rank test: $p=0.9105$, $p=0.4659$, $p=0.9033$.

(C) Kaplan-Meier survival curve of MNs survival following Ganetespib or DMSO control. Three independent differentiations separated. 50 cells per condition per experiment, Mantel-cox log-rank test: $p=0.0137$, $p=0.0372$, $p=0.0403$.

(D) Heat map of hierarchically clustered (k-means) of average relative protein abundance for each group from TMT-MS. N = 4 independent differentiations per condition.

bioRxiv preprint doi: https://doi.org/10.1101/2024.12.01.626216; this version posted December 1, 2024. The copyright holder for this preprint (which was not certified by peer review) is the author/funder, who has granted bioRxiv a license to display the preprint in perpetuity. It is made available under aCC-BY 4.0 International license.

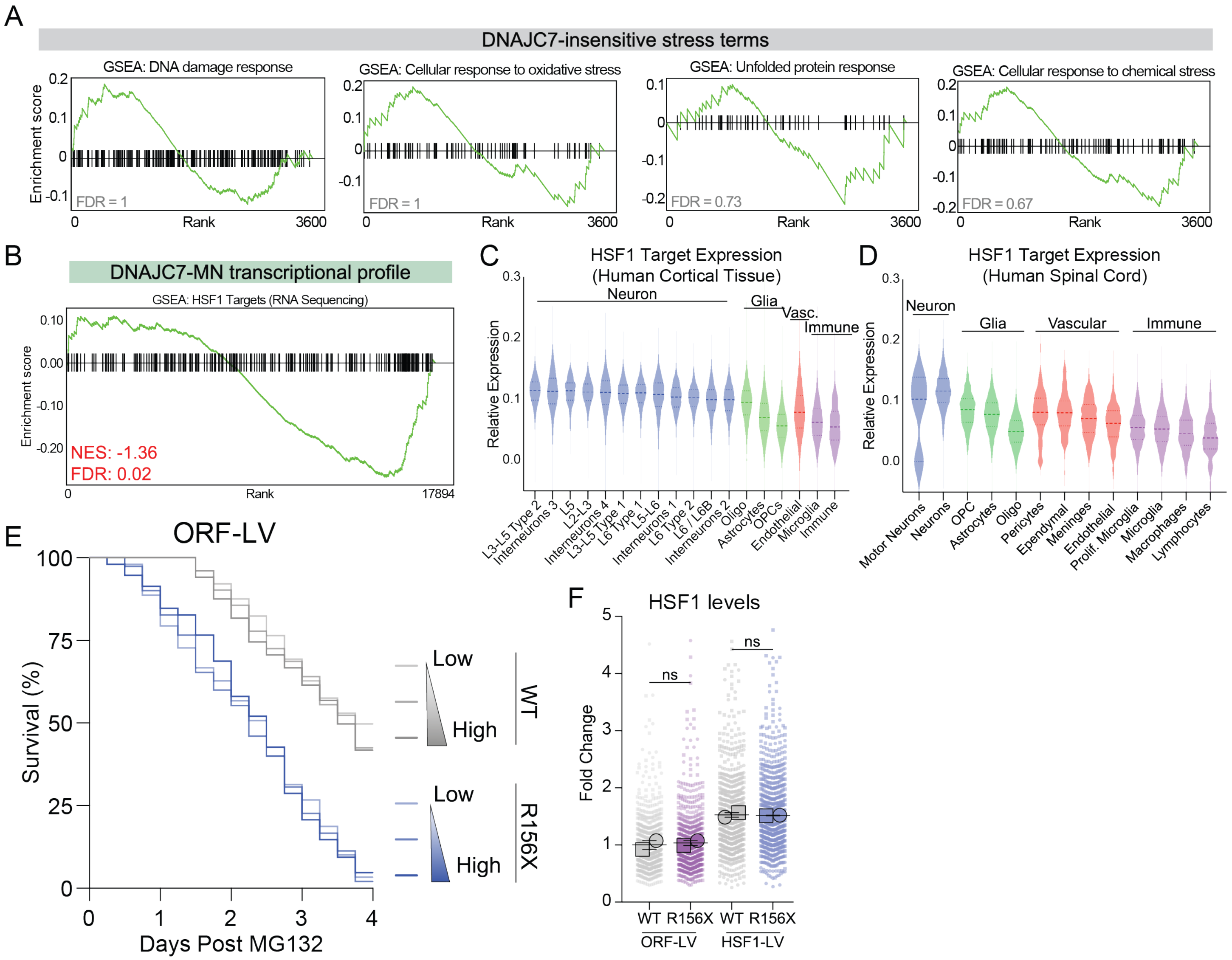

Figure S4. DNAJC7 regulates stress-induced activation of HSF1 and activation of HSF1 rescues the sensitivity of mutant DNAJC7-MN to stress

(A) GSEA of non-enriched “stress” related pathways in baseline proteomic dataset. DNA damage response (MSigDB#: M13636), unfolded protein response (MSigDB#: M5922), cellular response to oxidative stress (MSigDB#: M45123), and cellular response to chemical stress (MSigDB#: M29264).

(B) GSEA of HSF1 transcriptional targets (MSigDB#: M19734) de-enrichment in RNA transcriptional profile of DNAJC7-MNs. Kolmogorov–Smirnov test: NES = -1.36, FDR = 0.02.

(C and D) Violin plots of relative expression of HSF1 transcriptional targets (MSigDB#: M19734) as a group in human cortical (left) or spinal (right) tissue. Subtypes are categorized into Neuron, Glia, Vascular (Vasc.), or Immune cellular subgroups.

(E) Kaplan-Meier survival curve of MNs survival following MG132 with ORF-LV escalating doses of ORF-LV. 36-50 cells tracked per condition.

bioRxiv preprint doi: https://doi.org/10.1101/2024.12.01.626216; this version posted December 1, 2024. The copyright holder for this preprint (which was not certified by peer review) is the author/funder, who has granted bioRxiv a license to display the preprint in perpetuity. It is made available under aCC-BY 4.0 International license.

(F) Superplots of relative expression of HSF1 following HSF1-LV or ORF-LV transduction. Experiments are represented by distinct shaped symbols, individual cell values plotted in background. N = 2, Sidak's multiple comparisons test: WT vs R156X (ORF) p=0.9898, WT vs R156X (HSF1) p=0.9718.

bioRxiv preprint doi: https://doi.org/10.1101/2024.12.01.626216; this version posted December 1, 2024. The copyright holder for this preprint (which was not certified by peer review) is the author/funder, who has granted bioRxiv a license to display the preprint in perpetuity. It is made available under aCC-BY 4.0 International license.

SUPPLEMENTARY ITEMS

Table S1. Results of LC-MS/MS DNAJC7 IP vs IgG IP

Alternate ID	Log2FC	P Value
AASS	Inf	0.00532813
ACTC1	3.22706891	0.00185401
ACTG1	1.88535743	0.00056096
ACTR1A	Inf	0.00108562
ALB	-0.5986374	0.02876834
ALK	-0.5145732	0.41686553
ATP5F1A	Inf	4.1619E-05
ATP5F1B	Inf	0.08508565
ATP5F1C	Inf	0.00038817
ATP6V1H	Inf	0.00749043
CAND1	Inf	0.03994197
COPA	Inf	0.05330059
CRMP1	Inf	0.03584222
DCD	-0.6166714	0.34141976
DCLK1	Inf	0.03994197
DCTN2	Inf	0.07982533
DCX	Inf	0.00048413
DNAJC7	Inf	0.02727733
DPYSL3	Inf	0.10173079
DSP	-0.3785116	0.63876821
DYNC1H1	6.10852446	0.18347201
EEF1A1	2.66675659	0.00420024
EEF1G	Inf	0.0013239
EEF2	Inf	0.06384816
EPPK1	Inf	0.00749043
FASN	Inf	0.00862393
GAP43	Inf	0.0079662
GAPDH	0.5849625	0.10620776
HBA1	-0.8171359	0.00377202
HBB	-0.5849625	0.070484
HBG1	-0.7484612	0.10639791
HIST1H2BL	-1.5849625	0.070484
HIST2H2AC	-1.0703893	0.10616646
HNRNPC	Inf	0.00658385
HNRNPD	Inf	0.01944177
HNRNPK	Inf	0.02696532
HNRNPL	Inf	0.00130546
HNRNPU	Inf	0.08279449
HPX	-1.8073549	0.05831181

bioRxiv preprint doi: https://doi.org/10.1101/2024.12.01.626216; this version posted December 1, 2024. The copyright holder for this preprint (which was not certified by peer review) is the author/funder, who has granted bioRxiv a license to display the preprint in perpetuity. It is made available under aCC-BY 4.0 International license.

HRNR	-0.334419	0.6638042
HSP90AB1	2.12338242	0.00713971
HSPA1A	Inf	3.8149E-05
HSPA8	3.66296501	0.0009402
HUWE1	Inf	0.050265
IGF2BP1	1.73696559	0.05723523
ILF2	Inf	0.00749043
ILF3	Inf	0.01007969
INA	4.7548875	0.00033753
JUP	-0.8744691	0.58228482
KHDRBS1	Inf	0.00219213
KIF3A	4.08746284	0.01288669
KPRP	-0.6166714	0.58106771
KRT1	-0.0412816	0.88158054
KRT10	-0.180938	0.45181364
KRT14	-0.7399372	0.05336884
KRT16	-0.6764609	0.14050629
KRT2	-0.2642126	0.26694695
KRT5	-0.2715964	0.16378998
KRT6A	-0.1554545	0.56959249
KRT8	Inf	0.00053253
KRT9	-0.3520601	0.18475469
LDHA	0.80735492	0.34864114
LRPPRC	Inf	0.21999967
MAP1B	Inf	0.00388254
MTHFD1	Inf	0.00377202
NEFL	Inf	0.0015411
NEFM	Inf	0.00121532
NONO	Inf	0.02242122
PFKP	Inf	0.11611652
PKM	Inf	0.14932117
PRPH	5.57742883	4.6256E-05
PRSS1	-0.3625701	0.72807179
SFXN1	Inf	0.00532813
SHROOM3	-0.6100535	0.15183454
SLC25A4	Inf	0.00452406
SLC25A6	Inf	0.0009402
TUBA1A	5.07201707	0.00256135
TUBA1B	5.57137344	0.00335634
TUBA4A	Inf	0.00149455
TUBB	3.57613865	0.00029025
TUBB2A	3.71933992	0.00023504
TUBB3	4.61667136	0.00057774

bioRxiv preprint doi: https://doi.org/10.1101/2024.12.01.626216; this version posted December 1, 2024. The copyright holder for this preprint (which was not certified by peer review) is the author/funder, who has granted bioRxiv a license to display the preprint in perpetuity. It is made available under aCC-BY 4.0 International license.

TUBB4A	Inf	0.00064663
TUBB4B	Inf	0.00024815
TUBB6	Inf	0.00178996
VDAC3	Inf	0.05723523
VIM	Inf	1.7159E-05
VPS33A	Inf	0.11340392

METHODS

Cell culture conditions

Induced pluripotent stem cells (iPSCs) were grown on Matrigel (Fisher), maintained with mTeSR1 medium (Stemcell technologies), and passaged every 5 days using Accutase (Innovative Cell Technologies). All cultures were kept at 37C with 5% CO2 and were grown in the absence of antibiotics. Furthermore, all cell lines used were tested monthly to ensure the absence of mycoplasma throughout the duration of this study.

iPSC Models

Differentiation of lower motor neurons (MNs) from iPSCs were performed as previously described[29,78-80]. Briefly, ~70% confluent iPSCs were dissociated using Accutate to achieve single-cell suspension and replated with mTeSR1 medium supplemented with 10μM ROCK inhibitor (Y-27632, DNSK International) at a density of 1.2 million cells/well of a 6 well plate. The next day, the mTeSR1 was removed and replaced with N2B27 differentiation medium (base of 50% Neurobasal and 50% DMEM:F12, supplemented with nonessential amino acids, GlutaMAX, N2, and B27; Gibco), further supplemented with small molecules: 10 μM SB431542 (DNSK International), 100 nM LDN-193189 (Tocris), 1 μM retinoic acid (RA; Sigma), and 1 μM Smoothened Agonist (SAG; DNSK International). This medium was maintained and changed daily until day 6 in culture, and subsequently replaced with N2B27 differentiation medium supplemented with 1 μM RA, 1 μM SAG, 5 μM DAPT (Tocris), and 4 μM SU5402 (DNSK International) to generate postmitotic spinal MNs. These cultures were then fed on a daily basis until day 14 in culture, and next were dissociated using TrpLE Express (Gibco) supplemented with DNase I (Worthington). MNs were then plated directly on Matrigel (BD Biosciences) coated cell culture plates and growth in NBM (base of neurobasal medium supplemented with nonessential amino acids, GlutaMAX, N2, B27, ascorbic acid (0.2 μg/ml, Sigma), brain-derived neurotrophic factor (BDNF), ciliary neurotrophic factor (CNTF), and glial cell line-derived neurotrophic factor (GDNF) (10 ng/ml, R&D Systems). For experiments requiring imaging or survival analysis, MNs were plated initially onto pre-coated Matrigel surfaces and allowed to attach for 24 hours, and the following day primary mouse glia cells (harvested from P0 mixed male and female pups of the CD1 strain as described previously[80]) were plated on top of MNs.

Coimmunoprecipitation followed by mass spectrometry or WB

MNs lysates were collected in IP buffer (10mM Hepes [pH 7.6], 100mM NaCl, 1mM dithiothreitol, 10% glycerol, 1% sodium deoxycholate, 0.1% SDS, 1% Triton X-100, 1x protease inhibitor cocktail, and 1x phosphatase inhibitor cocktail. Insoluble material from cell extracts was then cleared by centrifugation, and protein

bioRxiv preprint doi: https://doi.org/10.1101/2024.12.01.626216; this version posted December 1, 2024. The copyright holder for this preprint (which was not certified by peer review) is the author/funder, who has granted bioRxiv a license to display the preprint in perpetuity. It is made available under aCC-BY 4.0 International license.

concentrations were determined with a BCA kit (Pierce). Endogenous DNAJC7 was immunoprecipitated from 1mg of protein with anti-DNAJC7 antibody (Abcam, ab179830). IP of the antigen was conducted using Dynabeads Protein A magnetic beads (Invitrogen) following the manufacturer's protocol. *For preparation of mass spectrometry (MS)*: eluted IP'd material was purified for compatibility to MS detection by briefly being run through precast polyacrylamide gel (Bio-Rad) to embed the material, followed by cutting out entire gel stack and subject to further processing for MS-based proteomics. *For preparation of western bot*: eluted proteins were separated by SDS-PAGE and subsequent transfer to nitrocellulose membrane (Bio-Rad). Membranes were then blocked in tris-buffered saline (TBS) + 0.1% Tween 20 (Bio-Rad) + 5% nonfat dry milk (LabScientific) and subject to overnight incubation at 4ºC primary antibodies: DNAJC7 (1:1000, Abcam), HSP90 (1:500, Santa Cruz), MATR3 (1:10,000, Abcam), HSPA1A (1:1000, Novus), HNRNPU (1:500, Santa Cruz), and HNRNPK (1:000, Cell Signaling). All primary antibodies were diluted in TBS + 0.1% Tween + 5% nonfat dry milk. After multiple washes with TBS + 0.1% Tween, membranes were incubated with their corresponding secondary anti-mouse and anti-rabbit HRP-conjugated antibodies (1:5000, LI-COR Biotechnology). Membranes were then exposed to SuperSignal Pico chemiluminescent (Thermo Fisher Scientific) and imaged by ChemiDoc XRS+ system (Bio-Rad).

MN survival tracking

For MN survival experiments, lentiviral *SYN1-GFP* (PZ196, Addgene) was added to MNs in suspension prior to seeding onto ImageLock 96 well plates (Sartorius) precoated with Matrigel. MNs were then plated in NBM supplemented with neurotrophic growth factors BDNF, CNTF, GDNF, and AA with SYN1-GFP virus for 24 hours before undergoing a full medium change to remove virus. In the case of lentiviral rescue experiments, LV-ORF/HSF1/BAG3 were co-transduced with SYN1-GFP at this time. Following the 24-hour incubation period, viral medium are removed and replaced with fresh medium and primary mouse glial cells. MN medium is then replenished every 2 days. After 7 days (21 days in culture), MNs are then treated individually with one of several variety of pharmacological agents including DMSO control (Sigma), MG132 (Calbiochem), Ganetespib (Mendillo Lab via Selleckchem), Brefeldin (Mendillo Lab via InvivoGen), Direct Targeted HSF1 InhiBitor (DTHIB, Mendillo Lab via Medchem) supplemented with cell death indicator propidium iodide (1:5000, Sigma). MN survival was then tracked with live imaging using IncuCyte S3 system (Sartorius) for green (GFP) and red (PI) signal emitted from cells. Image analysis was performed using Fiji software (U. S. National Institutes of Health, Bethesda, Maryland, USA), where cells were marked as dead when PI displays focal accumulation in the nucleus.

Lentivirus production

To produce viruses (SYN1-GFP, LV-HSF1 [VectorBuilder], LV-ORF [VectorBuilder], LV-BAG3 [VectorBuilder]), HEK-293 cells were co-transfected with individual target lentivirus plasmids combined with packaging plasmids pMD2.G and psPAX2 vectors with HilyMax (Dojino Molecular Technologies). Virus was then collected from the cell medium 72 hours after transfection, filtered through a 0.22 µm PVDF syringe filter, and concentrated by centrifugation at 25,000g for 2 hours at 4ºC. Concentrated virus was then resuspended in neurobasal medium (Gibco), aliquoted in appropriate volumes, and stored at -80ºC.

bioRxiv preprint doi: https://doi.org/10.1101/2024.12.01.626216; this version posted December 1, 2024. The copyright holder for this preprint (which was not certified by peer review) is the author/funder, who has granted bioRxiv a license to display the preprint in perpetuity. It is made available under aCC-BY 4.0 International license.

Co-immunoprecipiation mass spectrometry

Mass spectrometry sample preparation: Proteins were submitted as gel bands. Bands were destained by successive washes in acetonitrile and 100 mM ammonium bicarbonate. Disulfide bonds were reduced by incubating in 20 mM dithiothreitol (30 minutes at room temperature). Resulting free thiols were capped by incubating in 50 mM iodoacetamide (30 minutes at room temperature protected from light). Bands were then washed in 100 mM ammonium bicarbonate prior to addition of 2 µg of trypsin enzyme (Promega) followed by overnight incubation at 37 °C. The following day, the digestion was halted by acidification with 20 % formic acid and the peptide solution was dried in a vacuum centrifuge. The samples were re-suspended in 30 µl of 0.1 % formic acid, sonicated for 5 minutes to fully dissolve, then benchtop centrifuged. The supernatant was transferred to a glass vial and placed in the cooled auto-sampler rack.

LC-MS/MS analysis: Peptides were analyzed by LC-MS/MS using a Dionex UltiMate 3000 Rapid Separation LC system coupled to a linear ion trap—Orbitrap hybrid Elite mass spectrometer (Thermo Fisher Scientific, San Jose, CA). Four-microliter peptide samples were loaded onto the trap column, which was 150 µm × 3 cm in-house packed with 3 µm ReproSil-Pur beads (New Objective, Woburn, MA). The analytical column was a 75 µm × 10.5 cm PicoChip column packed with 3-µm ReproSil-Pur beads. Solvent A was 0.1 % aqueous formic acid and solvent B was 0.1 % formic acid in acetonitrile. Peptides were separated on a 120-minute analytical gradient from 5 % to 40 % solvent B at a flow rate of 300 nl/min. The mass spectrometer settings included positive data-dependent acquisition (DDA) mode, a 2.40 kV source voltage, and 275 °C capillary temperature. MS1 scans were acquired from 400 to 2000 m/z at 60,000 resolving power and 1E6 automatic gain control in the orbitrap. The top fifteen most abundant precursor ions in each MS1 scan were selected for fragmentation with an isolation width of 1 Da. Collision-induced dissociation (CID) at 35 % normalized collision energy in the ion trap was applied for fragmentation. Previously selected ions were dynamically excluded from re-selection for 60 seconds. A value of 3E5 was set for the MS2 automatic gain control.

Mass spectrometry data analysis: Raw files were converted to mgf format and analyzed using the Mascot search engine (Matrix Science, London, UK. version 2.7). MS/MS spectra were searched against the SwissProt human database (2021 version). All searches included carbamidomethyl cysteine as a fixed modification and oxidized methionine, deamidated asparagine and glutamine, and acetylated N-terminal as variable modifications. Three missed tryptic cleavages were allowed. Mass tolerances of 10 ppm (MS1 precursor) and 0.6 Da (MS2) were applied. Results were imported into Scaffold 5 software (Proteome Software, Portland, USA) for visualization.

Tandem-mass-tag (TMT)-mass spectrometry

TMT- MS Sample Preparation: TMT-MS sample preparation was performed as previously described[66,81]. Briefly, 200 µg of whole cell extracts were methanol-chloroform precipitated. Extracted protein was resuspended in 6M guanidine in 100 mM TEAB and further reduced of disulfide bonds with DTT, followed by alkylation of cysteine residues with IAA. Proteins were then digested overnight at 37°C with 3 µg Trypsin/LysC (Promega). The digest

bioRxiv preprint doi: https://doi.org/10.1101/2024.12.01.626216; this version posted December 1, 2024. The copyright holder for this preprint (which was not certified by peer review) is the author/funder, who has granted bioRxiv a license to display the preprint in perpetuity. It is made available under aCC-BY 4.0 International license.

was then acidified with formic acid and desalted (C18 HyperSep columns). Peptides were resuspended in 100mM TEAB and100µg for used for each respective isobaric TMT tag. After a 75 min incubation at RT, the reaction was quenched with 5% (v/v) hydroxylamine to 0.3%. Isobarically labeled samples were then combined 1:1:1:1:1:1:1:1:1:1:1:1:1:1:1:1 and subsequently desalted. The sample was then fractionated using high pH reversed-Phase columns (Pierce) and dried before reconstituted in LC-MS Buffer A (5% acetonitrile, 0.125% formic acid) for LC-MS/MS analysis.

TMT-MS Data Collection: TMT-MS analysis was performed as previously described[66,81]. Briefly, samples were resuspended in 20 µl Buffer A (5% acetonitrile, 0.125% formic acid) and 3µg of each fraction was loaded for LC-MS analysis. Orbitrap Fusion was used to generate MS data. The chromatographic run was performed with a 4h gradient as previously described[66,81]. In MS3, the top ten precursor peptides were selected for analysis were then fragmented using 65% HCD before orbitrap detection. A precursor selection range of 400–1200 m/z was chosen with mass range tolerance. An exclusion mass width was set to 18 ppm on the low and 5 ppm on the high. Isobaric tag loss exclusion was set to TMT reagent. Additional MS3 settings include an isolation window = 2, orbitrap resolution = 60 K, scan range = 120 – 500 m/z, AGC target = 6*105, max injection time = 120 ms, microscans = 1, and datatype = profile.

TMT-MS Data Analysis and Quantification: TMT-MS data analysis was performed as previously described [66,81]. In brief, protein identification, TMT quantification, and analysis were performed with The Integrated Proteomics Pipeline-IP2 (Integrated Proteomics Applications, Inc., http://www.integratedproteomics.com/). Proteomic results were analyzed with ProLuCID, DTASelect2, Census, and QuantCompare. MS1, MS2, and MS3 spectrum raw files were extracted using RawExtract 1.9.9 software (http://fields.scripps.edu/downloads.php). Fully and half-tryptic peptide candidates were included in search space, all that fell within the mass tolerance window with no miscleavage constraint, assembled and filtered with DTASelect2 (ver. 2.1.3). Static modifications at 57.02146 C and 304.2071 K at N-term were included. Minimum peptide number was 2. The target-decoy strategy was used to verify peptide probabilities and false discovery ratios [82]. Minimum peptide length of six was set for the process of each protein identification and each dataset included a 1% FDR rate at the protein level based on the target-decoy strategy and Isobaric labeling analysis was established with Census 2 with no intensity threshold applied.

CRISPR/Cas9 editing

Healthy control iPSC line CS0002 was purchased from Cedar Sinai. iPSCs were edited by Applied StemCell Inc. (Milpitas, CA) and described in *Simkin et al*[83]. Briefly, one million iPSCs were electroporated with a mixture of sgRNA and Cas9 in a ribonucleoprotein format and ssODN. A small, presumably mixed population was then subjected to PCR and Sanger sequencing analysis. Once the heterogenous culture displayed sufficient repair with qualified HDR, the cells were then single-cell cloned. Individual colonies were picked after 2 weeks in culture and expanded. Positive clones were then further expanded and resequenced to confirm correct genotype prior to being cryopreserved and shipped. Furthermore, in previous work, *Simkin et al* performed extensive quality control on all iPSC clones utilized in this study including genomic DNA PCRs and Sanger sequencing, genomic

bioRxiv preprint doi: https://doi.org/10.1101/2024.12.01.626216; this version posted December 1, 2024. The copyright holder for this preprint (which was not certified by peer review) is the author/funder, who has granted bioRxiv a license to display the preprint in perpetuity. It is made available under aCC-BY 4.0 International license.

integrity and pluripotency assays, analysis of off-target Cas9 sites, and quantitative genotyping PCR-based copy number assays[84].

Soluble/Insoluble fractionation followed by WB

Protocol is adapted and modified from *Tsioras et al*[39]. Briefly, MNs lysates were collected in RIPA buffer (50mM Tris (pH 7.4), 150mM NaCl, 0.5% sodium deoxycholate, 0.2% SDS, 1% Triton X-100, 1x protease inhibitor cocktail, and 1x phosphatase inhibitor cocktail), sonicated 3 x 3s, 40V output (QSonica, LLC), and centrifuged at 20,000g for 20 minutes. The supernatant, which is the RIPA-soluble fraction, was removed, while the RIPA-insoluble pellet remaining was further washed twice in RIPA lysis buffer with centrifugations of 20,000g for 30 minutes were performed between washes. Following the final wash, the insoluble pellet was resuspended in 2x Laemmli Sample Buffer (Bio-Rad) and sonicated 3 x 5s at 70V output. Both soluble and insoluble fractions were then boiled at 95 ºC prior to loading for SDS-PAGE and WB analysis and probed for anti-HNRNPU (1:500, Santa Cruz), anti-HNRNPL (1:5000, Novus), and anti-MATR3 (1:10000, Abcam).

RNA Extraction

Cells were harvested in 1mL TRIzol Reagent (Thermo Fisher Scientific) per 1 million cells. 0.2mL of chloroform was mixed with the samples for 3 minutes prior to being centrifuged at 12,000g at 4ºC for 15 minutes. The RNA containing aqueous phase was then transferred to a new tube and 5 µg of Glycogen carrier (Thermo Fisher Scientific) was added to facilitate complete precipitation. Then, 0.5mL of isopropanol was added to the mixtures and left to incubate at room temperature for 25 minutes prior to being centrifuged for 10 minutes at 15,000g at 4ºC.

RNA Seq data processing

RNA libraries were sent to Novogene for QC, and library preparation (250~300 bp insert strand nonspecific library with polyA enrichment). Libraries were sequenced using the Illumna NovoSeq platform, targeting at least 80M 150bp paired-end reads per sample Raw reads were trimmed using cutadapt (v3.4) to remove Illumina universal adapter sequences (-aAGATCGGAAGAGCACACGTCTGAACTCCAGTCA,-AAGATCGGAAGAGCGTCGTGTAGGGAAAGAGTGT), trailing N bases (--trim-n), and bases with Phred score < 10 (--nextseq-trim=10), as well as any reads that were too short after trimming (-m 25). Trimmed reads were aligned to Gencode V38 (GRCh38.p13) with STAR (v.2.7.5a), using the flag –twopassMode Basic and default settings. Read counts were quantified at the gene-level using featureCounts (v2.0.1), specifying -s 0 for unstranded libraries.

Differential Expression

Gene-level differential expression analysis was performed in R with DESeq2 (v1.38.3). Lowly expressed genes were filtered out before analysis – a gene must have had at least 0.5 counts per million reads in at least 3 samples in order to be retained. Significant genes were defined as having FDR<0.05 and log2(FoldChange)>=1.

bioRxiv preprint doi: https://doi.org/10.1101/2024.12.01.626216; this version posted December 1, 2024. The copyright holder for this preprint (which was not certified by peer review) is the author/funder, who has granted bioRxiv a license to display the preprint in perpetuity. It is made available under aCC-BY 4.0 International license.

Gene ontology, GSEA, and STRING analysis

Gene ontology (GO) analysis was performed using The Database for Annotation, Visualization and Integrated Discovery (DAVID) [85,86]. Top terms were filtered by a statistical and fold enrichment cutoff and GraphPad Prism 10 was used to visualize the enriched GO enrichment. Gene set enrichment analysis (GSEA) was performed using GSEA-MSigDB software (v4.3.3)[87,88]. Standard parameters were used in the query but briefly are as follows: stress terms gene set database (49 queries), 10,000 phenotype permutations, platform: MSigDB.v2024.1.Hs.chip, weighted Signal2Noise ranking, meandiv normalization mode, and Wald statistic values (stats) from DESeq2 values from differential expression were used as ranking values for the genes. Kolmogorov–Smirnov test used to compute normalized enrichment score and false discovery rate correction. For STRING analysis, physical subnetwork analysis, where thickness of line represents confidence (scaled 0 to 1) with manual color annotation overlaid to corresponding relevant GO terms.

HSF1 target enrichment in single cell data sets

Processed single cell data sets from cortical and spinal tissues were downloaded from Synapse (syn45351388) and GEO (GSE190442), respectively. Author-provided counts and cell type annotations were used to create individual Seurat objects for cortex and spine data sets, and counts data were normalized using the *NormalizeData()* function. Following normalization, expression of HSF1 targets (from the HSF1_01 MSigDb gene set) at a per-cell were determined using the *AddModuleScore()* function. Module scores of HSF1 targets were then visualized for each cell type. For pseudobulk analysis in each cell type, counts were aggregated for individual patients using the Seurat function AggregateExpression. Aggregated counts for each cell type were provided to DESeq2 for differential expression analysis between ALS patients and healthy controls. For GSEA analysis, genes were ranked by their DESeq2 test statistic and analyzed for HSF1 target enrichment as described previously.

Immunocytochemistry

Cells were fixed with 4% paraformaldehyde (PFA) and blocked for 1 hour in phosphate-buffered saline (PBS) supplemented with 10% normal donkey serum (Jackson ImmunoResearch) and 0.1% Triton X-100. Cells were then incubated overnight at 4ºC with primary antibodies: ISL1/2(1:100, DSHB), MAP2 (Abcam, 1:5000), and HSF1 (ProteinTech, 1:200). Primary antibodies were removed and washed several times with PBS + 0.1% Triton before being incubated with Hoeschst 33342 (Invitrogen) and the appropriate secondary antibodies conjugated to Alexa Fluor 488, Alexa Fluor 555, or Alexa Fluor 647 fluorophores (1:500, Thermo Fisher Scientific) for 2 hours at room temperature. After several more washes in PBS + 0.1% Triton, cells were imaged directly in 24-well #1.5 glass bottom coated tissue culture plates (Cellvis) or coverslips were mounted with Fluoromount-G (Thermo Fisher Scientific).

RT-qPCR

bioRxiv preprint doi: https://doi.org/10.1101/2024.12.01.626216; this version posted December 1, 2024. The copyright holder for this preprint (which was not certified by peer review) is the author/funder, who has granted bioRxiv a license to display the preprint in perpetuity. It is made available under aCC-BY 4.0 International license.

Following RNA extraction, first-strand cDNA was synthesized from 1-2 µg of DNase I (Invitrogen) treated RNA using SuperScript IV reverse transcriptase (Thermo Fisher Scientific) and oligo dT primers following manufacturer's instructions. Synthesized cDNA was first diluted 1:10-1:20 and 2uL of diluted cDNA was used in each RT-PCR reaction performed with SYBR green (Thermo Fisher Scientific) on CFX system (Bio-Rad). PCR was performed under the following conditions: 95ºC for 3 min, 40 cycles at 95ºC for 10 sec and 60ºC for 30 sec, and final step from 65ºC to 95ºC in increments of 0.5ºC every 5 sec. All reactions were performed in duplicate. Average cycle of threshold (Ct) value of housekeeping gene GAPDH was subtracted from the Ct value of the gene of interest to obtain the ΔCt. Relative gene expression was then defined as the $2^{-\Delta Ct}$ (ΔΔCt) and normalized to each indicated sample control indicated in each experiment. Primer sequences used in this study: HSP70_F, ACCTTCGACGTGTCCATCCTGA; HSP70_R, TCCTCCACGAAGTGGTTCACCA; GAPDH_F, ACAACTTTGGTATCGTGGAAGG; GAPDH_R, GCCATCACGCCACAGTTTC.

Statistical Analysis

We performed all statistical analysis using Prism 9 software (GraphPad) and Fiji ((https://imagej.nih.gov/ij/). All values in figures with error bars are presented as mean ± standard error of the mean (SEM). We classified an independent biological replicate as an independent iPSC differentiation performed on a different day. All numbers (*n*), significance values (*P* or *Q* value), and statistical test performed are specified either in the Results section or in each specific corresponding figure legend. To ensure that pooling of data in each experiment was appropriate, we first tested whether datasets variances were significantly different using Brown-Forsythe analysis of variance (ANOVA) test. Following this, we next used the D'Agostino-Pearson test to test whether sample data fit a Gaussian distribution. For experiments that included at least n = 3, we performed one-way ANOVA followed by Tukey's post hoc for parametric test, and Kruskal-Wallis rank test with Dunn's correction for multiple comparisons for nonparametric test). All survival experiments were performed at least three times, with equal numbers of neurons from each replicate being used for quantification shown. Statistical analysis was then performed using a two-sided Mantel-Cox log-rank test from 40-50 neurons per replicate per experiment. All TMT-based proteomics experiments were analyzed with Bayesian analysis of variance using BAMarray 3.0, a Java software package that implements the Bayesian ANOVA for microarray (BAM) algorithm. The BAM approach uses a special type of inferential regularization known as spike-and-slab shrinkage, which provides an optimal balance between total false detections (the total number of genes falsely identified as being differentially expressed) and total false nondetections (the total number of genes falsely identified as being nondifferentially expressed)[89]. Details statistics for each Figure described below:

- Figure 1: (C) n = 3 experiments were from 3 independent differentiations of either DNAJC7 IP or negative control IgG IP. (D) individual q values from all enriched terms are as follows: Reactome, from top to bottom: 2E-11, 0.00057, 0.00062; Molecular Function, from top to bottom: 0.0019, 0.0028, 0.0075.

bioRxiv preprint doi: https://doi.org/10.1101/2024.12.01.626216; this version posted December 1, 2024. The copyright holder for this preprint (which was not certified by peer review) is the author/funder, who has granted bioRxiv a license to display the preprint in perpetuity. It is made available under aCC-BY 4.0 International license.

- Figure 2: (C) n = 4 independent differentiations; values represent the mean ± standard error of the mean (SEM), unpaired t test (two-tailed): **** P<0.0001. (E) n = 6 differentiations; values represent the mean ± min/max. unpaired t test (two-tailed): ns = 0.276, **** P<0.0001.

- Figure 3: (D) 150 cells tracked per condition, Mantel-Cox log-rank test: p<0.0001. (E) 129 cells tracked per condition, Mantel-Cox log-rank test: p=0.8085. (F) 150 cells tracked per condition, Mantel-Cox log-rank test: p=0.0001. (L) individual q values from all enriched terms are as follows: Reactome, from top to bottom: 0.000022, 0.000029, 0.0017.

- Figure 4: (D) n = 3 independent differentiations; values represent the mean ± standard error of the mean (SEM), Unpaired t test (two-tailed): CRYZ ***P=0.0002, HSPB1 ****P<0.0001. (G) n = 3 independent differentiations; values represent the mean ± standard error of the mean (SEM), Two-way ANOVA: *p<0.05, **p<0.01, ***p<0.001. (H) n = 2-3 independent differentiations; values represent the mean ± standard error of the mean (SEM), Two-way ANOVA: *p<0.05. (I) 144 cells tracked per condition, Mantel-Cox log-rank test: WT vs R156X (10 μM) p<0.0001, WT vs R156X (20 μM) p<0.0001. (J) 150 cells tracked per condition, Mantel-Cox log-rank test: WT+ORF vs R156X+ORF p<0.0001, R156X+HSF1 vs R156X+ORF p<0.0001, R156X+HSF1 vs WT+HSF1 p=0.177.

Data Availability

The mass spectrometry proteomics data have been deposited to the MassIVE repository with the identifier: (MSV000095891). Further information and requests for resources and reagents should be directed to and will be fulfilled by the Lead Contact, Evangelos Kiskinis pending scientific review and a completed material transfer agreement. Requests for these items should be submitted to: evangelos.kiskinis@northwestern.edu.
